# Microsurgery Versus Radiosurgery for Vestibular Schwannoma: Which Approach Yields Better Long-Term Results?

**DOI:** 10.7759/cureus.98470

**Published:** 2025-12-04

**Authors:** Mariana Olvera Morales, Isabela Morales Castillo, Jorge Pelayo Fernández, Olga N Polania Pérez, José A Robles Illescas, Alhy N Villar Vilchis, Edna D Valdez Mendoza, Jorge F Ducoing Castillo, Jael J Frank Nuñez, Miriam A Ortiz Hernández, Marco A Sánchez Fernández, Daniela A Ramirez Molina, Carlos M Fuentes Reyes, Jose R Flores Valdés

**Affiliations:** 1 General Medicine, Universidad Autónoma de Guadalajara, Guadalajara, MEX; 2 General Medicine, Universidad Anáhuac Querétaro, Santiago de Querétaro, MEX; 3 General Practice, Universidad La Salle, Ciudad de México, MEX; 4 General Practice, Universidad de Santander, Bucaramanga, COL; 5 General Medicine, Universidad Nacional Autónoma de México, Ciudad de México, MEX; 6 General Practice, Universidad Autónoma del Estado de Morelos, Cuernavaca, MEX; 7 General Medicine, Universidad para el Bienestar Benito Juárez García, San José Iturbide, MEX; 8 General Practice, Universidad Juárez del Estado de Durango, Durango, MEX; 9 General Practice, Universidad Popular Autónoma del Estado de Puebla, Puebla de Zaragoza, MEX; 10 General Medicine, Universidad de la Salud del Estado de México, Toluca de Lerdo, MEX; 11 General Practice, Tecnológico de Monterrey, Monterrey, MEX; 12 General Medicine, Universidad Veracruzana, Xalapa, MEX; 13 Thoracic Head and Neck Medical Oncology, MD Anderson Cancer Center, Houston, USA

**Keywords:** acoustic neuroma, hearing loss, radiosurgery, surgical procedures, tinnitus, vestibular schwannoma

## Abstract

Vestibular schwannoma (VS) is a common benign intracranial tumor that can significantly affect quality of life (QoL), particularly through hearing loss and balance impairment. Microsurgery and radiosurgery are established treatment options, but there is ongoing debate over which provides better long-term outcomes, especially for tumors smaller than 3 cm.

This systematic review aims to compare the long-term outcomes of microsurgery versus radiosurgery for the management of VS ≤ 2.5 cm.

We conducted a systematic review of cohort studies involving patients aged 18 years or older with VS measuring ≤ 2.5 cm, comparing outcomes of microsurgery and radiosurgery. Relevant databases were searched, and studies were selected and assessed for methodological quality. Tumor control, hearing preservation, and improvement in vestibular symptoms were examined. A thorough database search identified 547 articles, of which 4 met the inclusion criteria and were selected for analysis, encompassing a total of 1,167 participants.

Microsurgery showed superior tumor control and greater improvement in vestibular symptoms, whereas radiosurgery was associated with higher rates of hearing preservation. However, findings varied across studies, and the absence of randomized controlled trials limits the strength of current evidence.

Both microsurgery and radiosurgery offer benefits depending on the patient's clinical priorities. Treatment decisions should be individualized. Further prospective, reliable studies are needed to determine the optimal management strategy based on long-term functional outcomes and QoL.

## Introduction and background

Vestibular schwannomas (VS), also known as acoustic neuromas, are benign tumors that develop from the Schwann cells of the vestibular branch of the eighth cranial nerve [1]. First described in 1777 during an autopsy, the first successful surgical removals were achieved in the late 19th century [2]. These tumors account for 85%-90% of intracranial growths arising at the cerebellopontine angle and are considered the third most common intracranial benign tumors, following meningioma and pituitary adenomas [3]. Most cases are unilateral, typically in patients aged 20 to 40 years. Bilateral and earlier presentation is associated with neurofibromatosis type 2 [3,4]. The clinical presentation includes hearing loss and persistent tinnitus, with additional symptoms such as vertigo, headache, visual changes, otalgia, and facial numbness. Large tumors have been linked to trigeminal and facial neuropathies, as well as brainstem compression and hydrocephalus [3,5].

Recent studies report an incidence of approximately 0.6-1.9 cases per 100,000 individuals per year, with higher rates among adults aged 65-74 and no significant sex difference. This increase may be attributed to improvements in detection methods, particularly the widespread use of magnetic resonance imaging (MRI) [6-8]. Risk factors associated with VS are noise exposure and high doses of ionizing radiation. Some studies have attempted to establish a link between the use of the telephone and VS, although the evidence remains inconclusive. Other potential risk factors studied include smoking, occupation, allergic rhinitis, asthma, and eczema. Smoking has also been evaluated, with more recent epidemiologic data suggesting a negative association rather than an increased risk [3,4].

Patients presenting with neurological or otological symptoms suggestive of a lesion in the cerebellopontine angle require both clinical evaluation and imaging studies. MRI, being the gold standard for investigation, plays a crucial role in excluding other lesions and aiding in initial treatment. Specifically, T1-weighted imaging with gadolinium contrast has demonstrated superior sensitivity and specificity compared to T2-weighted imaging without contrast. However, T2-weighted imaging without contrast has been associated with fewer adverse reactions and is more cost-effective for clinical practice [1,4].

Several treatment options are available, including conservative management, microsurgery, radiotherapy, and radiosurgery. The choice of the optimal treatment depends on factors such as the tumor’s location, size, growth rate, the patient's general health, hearing status, the surgeon's experience and comfort with a particular approach, and the overall goals of the operation [9-11]. When conservative treatment is selected, it typically involves regular MRI monitoring, initially scheduled every six months and then annually. If the tumor shows minimal growth, the interval may be extended every two years.

Observation presents certain challenges, particularly the high cost of MRIs and the potential for patient attrition during follow-up. However, the advantages of observation include the possibility of delaying or even avoiding the need for surgical or radio-surgical intervention.

Radiotherapy is primarily aimed at controlling tumor growth, while the objective of surgery and radiosurgery is to achieve complete tumor removal while preserving neurological function and hearing, when possible. Tumors smaller than 2.5 cm (Koos I-II) are particularly important clinically because they present the greatest uncertainty regarding whether microsurgery or radiosurgery offers the best long-term outcomes. In microsurgery, the retro-sigmoid approach has traditionally been the most used due to its popularity among neurosurgeons and its wide applicability, while the translabyrinthine and middle cranial fossa approaches are also commonly employed depending on tumor sizes and hearing status. However, the optimal treatment modality for small- to medium-sized tumors remains a matter of debate [12,13].

The following post-treatment complications have been reported: brainstem edema, intracranial infection, subcutaneous effusion, transient abducens, glossopharyngeal nerve palsy, and hemorrhage resulting from cerebellar and brainstem contusion complicated by edema. The case fatality rate associated with surgery is low. These complications were found to be correlated with tumor size, but not with the type of follow-up [1,11].

Although numerous studies have compared the available therapeutic options, uncertainty persists regarding whether microsurgery or radiosurgery is the most appropriate treatment to achieve the best outcomes. Some gaps remain concerning which treatment yields better results, as well as the short- and long-term prognosis for each approach.

Small vestibular schwannomas (<2.5 cm) are the most debated group because both microsurgery and radiosurgery are viable options. Long-term outcomes, commonly defined as follow-up longer than two years, remain inconsistently reported across studies. This systematic review aims to assess the long-term results of microsurgery versus radiosurgery in patients with vestibular schwannoma.

## Review

Methods

Search Strategy

The current study adhered to the guidelines set by the Preferred Reporting Items for Systematic Reviews and Meta-Analyses (PRISMA 2020), as well as the methodological framework outlined in the 10 Steps to Conduct a Systematic Review [14,15]. Both are available under the Creative Commons Attribution license.

**Figure 1:**
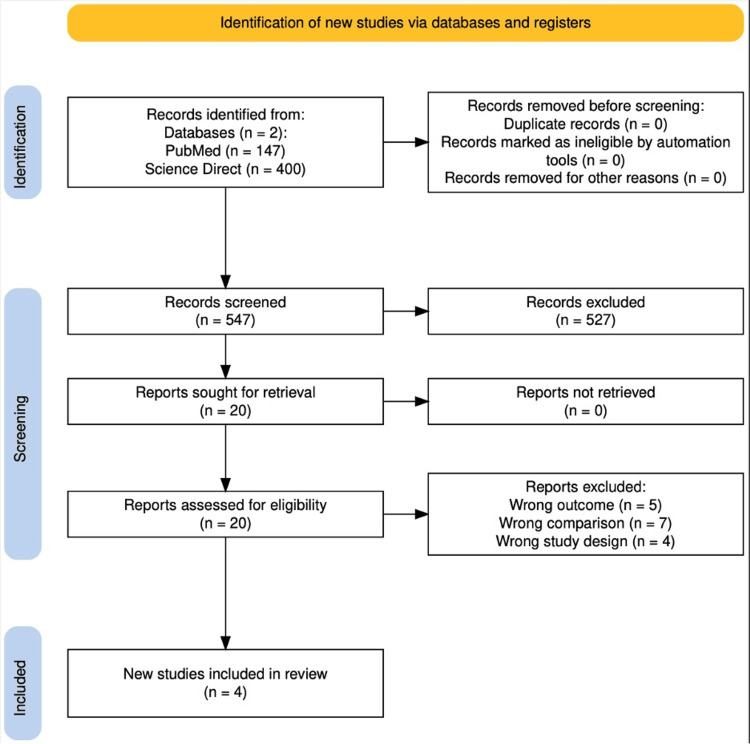
Preferred Reporting Items for Systematic Reviews and Meta-Analyses (PRISMA) flowchart of the study selection process A total of 547 records were screened following the PRISMA guidelines. Records were identified, screened, assessed for eligibility, and excluded based on predefined criteria. The reasons for exclusion included wrong outcome (n = 5), wrong comparison (n = 7), and wrong study design (n = 4).

This meticulous approach enabled the creation of a homogeneous dataset, facilitating a more accurate and reliable analysis of the results. We conducted a systematic literature review to assess and compare the effectiveness of microsurgery versus radiosurgery in patients with VS. The study aimed to evaluate which surgical approach yielded better long-term results. The review spanned the period from December 2002 to November 2023 and included databases such as PubMed and ScienceDirect. The final search was performed in July 2025. A comprehensive search strategy was employed using both Medical Subject Headings (MeSH) and free-text keywords, including “acoustic neuroma”, “vestibular schwannoma”, “surgical procedures”, “radiosurgery”, “hearing loss”, and “tinnitus”. Only studies evaluating tumors smaller than 2.5 cm were eligible for inclusion, as outcomes may differ significantly in larger VS. The size restriction was applied because functional outcomes and tumor-control rates vary significantly in tumors larger than 2.5-3 cm. The number of records retrieved from each database and the full selection process are detailed in Figure 1. 

Types of Study

This systematic review included observational comparative studies, primarily cohort designs that reported long-term outcomes in patients treated with microsurgery versus radiosurgery as treatment for VS. Three of the included studies were cohort studies, and one study was a prospective nonrandomized comparative design. All studies were published in English and conducted from December 2002 to November 2023.

We excluded case reports, case series, cross-sectional studies, dissertations, book chapters, protocol articles, reviews, news articles, conference abstracts, letters to the editor, editorials, and comment publications. Furthermore, we removed duplicate studies, those without a comprehensive methodology, and studies for which essential data were unavailable, as well as studies from which we did not receive a response from the author via email.

Types of Participants

Stringent criteria were established to include only high-quality studies. Eligible patients were adults over 18 years who were diagnosed with VS (<2.5 cm), treated exclusively with microsurgery or radiosurgery, and evaluated pre- and post-treatment using the Gardner-Robertson scale. Exclusion criteria included individuals who did not meet the age requirements, received alternative treatments, were pregnant, were pediatric patients, or lacked record data on treatment progression, complications, or outcomes.

Types of Interventions

This systematic review included studies that met the inclusion criteria in which VS was treated primarily with either microsurgery or radiosurgery. The objective was to compare the progression and determine which surgical approach yielded better long-term outcomes.

Pre- and post-treatment follow-up was essential, as it enabled the assessment of treatment effectiveness through clinical outcomes such as hearing preservation, tinnitus, and vertigo. These outcomes were used to compare the two types of interventions, microsurgery versus radiosurgery.

Studies were excluded if they involved participants under 18 years of age, tumors larger than 2.5 cm in diameter, alternative treatment modalities, or lacked pre- and post-treatment assessments. Additionally, studies were excluded if data on treatment evolution, complications, or outcomes were not reported.

Outcomes

The primary endpoint was to evaluate the hearing function after surgery using the Gardner-Robertson scale. This scale classifies hearing into five categories based on speech discrimination and pure-tone average, allowing consistent comparison of serviceable versus non-serviceable hearing. Secondary outcomes included improvements in vertigo and tinnitus variables assessed using the Dizziness Handicap Inventory and Tinnitus Handicap Inventory, which evaluated variables such as vertigo, tinnitus, and unsteadiness. Due to heterogeneity in the study design, outcome definitions, and follow-up durations, a qualitative synthesis was performed, and outcomes were compared descriptively across studies without conducting a meta-analysis.

Selection of Studies

Two reviewers (DAC, CAUM) independently screened titles and abstracts using Rayyan (Rayyan Systems Inc., Cambridge, MA, USA), a web-based tool designed to facilitate systematic review. A third independent reviewer (PMRB) then verified the relevance of the studies according to predefined inclusion and exclusion criteria. Full-text articles were subsequently assessed, where two reviewers (EGV, CAUM) independently evaluated eligibility. Any disagreements at this stage were resolved through consensus and with the assistance of the third review author (DRAF). Potentially eligible studies were assessed in detail against the criteria and either included or excluded accordingly.

For data extraction, two reviewers (MATA, PMRB) applied the specific criteria and guidelines of the selected tools. A standardized data extraction form was developed in Microsoft Excel (Microsoft Corp., Redmond, WA, USA) to systematically collect information on study characteristics, interventions, outcomes, and follow-up. Discrepancies were resolved through discussion with a third, blinded reviewer (DRAF). Tumor size was extracted as reported in each study (diameter in mm or cm, or Koos grading), and no unit conversions or standardizations were performed to avoid misclassification. Only data corresponding to tumors smaller than 2.5 cm were extracted and analyzed, even in studies that reported additional size categories, to ensure consistency with the predefined inclusion criteria. 

After applying these criteria, four observational comparative studies (three cohort studies and one prospective nonrandomized study) were included in the final review, encompassing a total of 1,167 participants. 

Assessment of Risk of Bias of Included Studies

Two reviewers (JDC, ONPP) independently evaluated the risk of bias in each study, following the specific criteria and guidelines of the respective tools. Inter-rater agreement between the two reviewers was high, and a formal Cohen's kappa statistic was not calculated because any discrepancies were resolved through discussion with a third reviewer (ANVV). The risk of bias assessment for the included studies in this systematic review was conducted using the Newcastle-Ottawa scale (NOS) [16], applying the cohort study version of the tool to all four observational studies, which evaluated quality across three domains: selection, comparability, and outcome. The total score reflected the overall quality of each study, with a maximum total score of 9. Analyzing Table 1 in detail, three cohort studies and one prospective nonrandomized comparative study achieved 4 points in the selection domain, which was the highest score, indicating an adequate selection method for observational designs. However, in the comparability domain, each study received only 1 point, suggesting limited capacity for control of variables. Finally, the outcome domain presented three studies scoring with 3 points, and one study scoring with 2 points, indicating slight variations in outcome assessment. Based on this evaluation, two cohort studies and the prospective nonrandomized study each scored a total of 8 points, while one cohort study scored 7 points, which indicated “good quality,” demonstrating a generally low risk of bias.

**Table 1:** Newcastle-Ottawa Tool for cohort studies’ risk of bias appraisal. Four observational studies were evaluated using the Newcastle-Ottawa Quality Assessment Scale (NOS). The overall quality of each study was categorized as good quality, fair quality, and poor quality: good-quality studies, 3 or 4 stars in the selection domain, 1 or 2 stars in the comparability domain, and 2 or 3 stars in the outcome/exposure domain; fair-quality studies, 2 stars in the selection domain, 1 or 2 stars in the comparability domain, and 2 or 3 stars in the outcome/exposure domain; and poor-quality studies, 0 or 1 star in the selection domain, 0 stars in the comparability domain, and 0 or 1 star in the outcome/exposure domain. Based on this evaluation, all four included studies were classified as of good quality.

Author/year	Study design	Selection	Comparability	Outcomes	Total	Subjective evaluation
Tatagiba et al. (2023) [17]	Cohort study	4	1	3	8	Good quality
Myrseth et al. (2009) [18]	Prospective nonrandomized study	4	1	3	8	Good quality
Karpinos et al. (2002) [19]	Cohort study	4	1	2	7	Good quality
Pollock et al. (2006) [20]	Cohort study	4	1	3	8	Good quality

Results

We systematically reviewed the literature to evaluate the effectiveness of microsurgery versus radiosurgery in treating VS. Available information was gathered through PubMed and ScienceDirect covering the period from December 2002 to November 2023.

The flowchart of the selection was made according to the PRISMA 2020 reporting guideline (Figure 1). As shown in the diagram, our initial search yielded 547 potential records. After removing duplicates and screening titles and abstracts, we assessed 20 full-text articles for eligibility. All eligible studies were retrieved. After exhaustive screening, 16 articles were excluded for wrong outcomes, wrong comparisons, and wrong study design, and finally, 4 studies were included in this systematic review [14].

The preselected observational studies were rigorously assessed using the NOS, an openly accessible tool available from the Ottawa Hospital Research Institute. The cohort-study version of the NOS was applied to all four studies, as each included two comparison groups followed over time. All four included studies were rated as good quality (100%), with no studies falling into the fair (0%) or poor quality (0%) categories. 

Results of individual studies

Tumor Control

Tatagiba et al. reported an incidence of recurrent tumor of 7% after microsurgical resection and 11% after stereotactic radiosurgery, with superior tumor control observed in the microsurgical resection in the Kaplan-Meier analysis (P = 0.031) [17]. Karpinos et al. demonstrated that there was no significant difference in tumor growth, reporting a 100% control rate in patients who underwent microsurgery versus 91% in those patients treated with radiosurgery [19]. Similarly, Pollock et al. reported 100% tumor control with microsurgery compared to 96% with radiosurgery, with no statistically significant difference reported [20].

Hearing Function

When comparing hearing outcomes following treatment, Tatagiba et al. found long-term post-interventional hearing loss in 54% of patients, corresponding to 63% of patients who underwent microsurgery, and 46% in those treated with radiosurgery. Hearing deterioration was similar between both treatment techniques for small VS (Koos grades I and II), but more pronounced in microsurgical resection of large VS (Koos grades III and IV) [17]. Myrseth et al. reported that 0% of the patients who underwent surgery had serviceable hearing postoperatively, while 13 out of 28 patients had serviceable hearing preoperatively. In contrast, 25 out of 60 patients who underwent gamma knife radiosurgery had serviceable hearing before treatment, and 17 of them (68%) had serviceable hearing two years after the procedure [18]. Karpinos et al. reported that radiosurgery was more effective than microsurgery in hearing preservation, with 57.5% in radiosurgery patients, compared to 14.4% in microsurgery patients [19]. Lastly, Pollock et al. showed that serviceable hearing was more frequent in the radiosurgery group after the last follow-up (63% versus 5%, P < 0.0001) compared to the microsurgical resection group [20].

Vertigo and Tinnitus

Tatagiba et al. reported that microsurgical resection more frequently improved tinnitus, vertigo, and imbalance compared to stereotactic radiosurgery, which was associated with a 12% incidence of worsening tinnitus [17]. Myrseth et al. reported no significant trend toward improvement or worsening in balance function in either treatment group; however, patients in the microsurgery group reported a reduction in tinnitus compared to patients treated with gamma knife radiosurgery after one year (P = 0.03) [18]. On the other hand, Karpinos et al. reported that patients in the radiosurgery group experienced significantly more tinnitus at the long-term follow-up compared to patients who underwent microsurgery (26.5% versus 0%, P = 0.04). However, no significant difference was noted in the incidence of worsened imbalance between the two groups (23.5% for radiosurgery versus 22.4% for microsurgery, P = 0.932) [19]. Finally, Pollock et al. reported that the radiosurgery group had lower Dizziness Handicap Inventory scores compared to the microsurgery group (16.5 versus 8.4, P = 0.02), although no significant difference was reported regarding tinnitus [20]. Supplementary data supporting the findings discussed above are provided in Table 2.

**Table 2:** General outcomes This table describes the general outcomes of the four studies comparing microsurgery and radiosurgery for VS. Results suggest similar tumor control for small tumors (Koos I-II), while surgery is superior for larger tumors (Koos III-IV). Radiosurgery shows better hearing preservation, whereas surgery improves tinnitus, vertigo, and trigeminal symptoms. CPA: cerebellopontine angle, G&R: Gardner and Robertson, H&B: House and Brackmann, N/A: not applicable, VS: vestibular schwannoma.

Author	Tatagiba et al. [17]	Myrseth et al. [18]	Karpinos et al. [19]	Pollock et al. [20]
Year	2023	2009	2002	2006
Title	A comparative study of microsurgery and gamma knife radiosurgery in vestibular schwannoma evaluating tumor control and functional outcome	Vestibular schwannoma: surgery or gamma knife radiosurgery? A prospective, nonrandomized study	Treatment of acoustic neuroma: stereotactic radiosurgery versus microsurgery	Patient outcomes after vestibular schwannoma management: a prospective comparison of microsurgical resection and stereotactic radiosurgery
Country	Germany	Norway	USA	USA
Study design	Retrospective bicentric cohort	Prospective nonrandomized study	Retrospective cohort	Prospective cohort
Females	502	50	29	36
Males	399	38	67	46
Intervention	Vestibular schwannoma microsurgery	Vestibular schwannoma microsurgery	Vestibular schwannoma microsurgery	Vestibular schwannoma microsurgery
Comparator	Radiosurgery	Surgery and gamma knife radiosurgery	Gamma knife radiosurgery	Radiosurgery
Total age, mean (SD)	54.47 (±13.70)	55	61.6	51.4
Cases' age, mean (SD)	47.45 (±12.48)	>20	62.5	48.2
Controls' age, mean (SD)	59.00 (±12.56)	N/A	44.8	53.9
No. of cases	342	88	23	36
No. of controls	559	N/A	96	46
Comorbidities	N/A	N/A	N/A	N/A
Tumor location	N/A	Cerebellopontine angle	Intracanalicular and cerebellopontine	Intracanalicular and cerebellopontine
Tumor size				
Small cases (mm/cm)	N/A	<15 mm	<2.0 cm	N/A
Medium cases (mm/cm)	N/A	N/A	2.0-3.9 cm	N/A
Large cases (mm/cm)	N/A	>20 mm	>4.0 cm	N/A
Small controls (mm/cm)	N/A	N/A	<2.0 cm	N/A
Medium controls (mm/cm)	N/A	N/A	2.0-3.9 cm	N/A
Large controls (mm/cm)	N/A	N/A	>4.0 cm	N/A
Koos I cases	28	N/A	N/A	N/A
Koos I controls	86	N/A	N/A	N/A
Koos II cases	82	N/A	N/A	N/A
Koos II controls	213	N/A	N/A	N/A
Koos III cases	130	N/A	N/A	N/A
Koos III controls	200	N/A	N/A	N/A
Koos IV cases	102	N/A	N/A	N/A
Koos IV controls	60	N/A	N/A	N/A
Cystic morphology cases	31	N/A	N/A	N/A
Cystic morphology controls	24	N/A	N/A	N/A
Preoperative clinical status cases				
H&B 1-2 cases	340	88	17	N/A
H&B 1-2 controls	546	N/A	58	N/A
G&R 1-2 cases	219	88	7	N/A
G&R 1-2 controls	247	N/A	17	N/A
Facial spasm cases	0	N/A	N/A	N/A
Facial spasms controls	0	N/A	N/A	N/A
Tinnitus cases	244	73	8	N/A
Tinnitus controls	417	N/A	35	N/A
Vertigo cases	206	42	15	N/A
Vertigo controls	343	N/A	47	N/A
Trigeminus cases	48	N/A	6	N/A
Trigeminus controls	46	N/A	12	N/A
Preoperative clinical status cases				
Follow-up (months)	N/A	88	46.7	N/A
H&B 1-2 cases	340	74	6	N/A
H&B 1-2 controls	546	N/A	38	N/A
G&R 1-2 cases	82	17	N/A	N/A
G&R 1-2 controls	134	N/A	N/A	N/A
Facial spasm cases	0	N/A	N/A	N/A
Facial spasms controls	29	N/A	N/A	N/A
Tinnitus cases	64	59	16	N/A
Tinnitus controls	200	N/A	44	N/A
Vertigo cases	91	8	10	N/A
Vertigo controls	268	N/A	3	N/A
Trigeminus cases	13	1	N/A	N/A
Trigeminus controls	41	0	N/A	N/A
Complication cases	46	N/A	2	N/A
Complication controls	62	N/A	3	N/A
CDC 2	21	N/A	N/A	N/A
CDC 3a	29	N/A	N/A	N/A
CDC 3b	12	N/A	N/A	N/A
CDC > 4	0	N/A	N/A	N/A
Mortality cases	0	N/A	N/A	N/A
Mortality controls	0	N/A	N/A	N/A
Key points	Tumor control is comparable in small VS, but surgery is superior for large VS	Facial nerve function, gamma knife radiosurgery, hearing preservation, microsurgery, quality of life, VS	Acoustic neuroma, stereotactic radiosurgery, microsurgery, gamma knife	Acoustic neuroma, outcome, radiosurgery, VS

Summary of findings

Across the four studies (n = 1,167 participants), microsurgery demonstrated higher tumor control rates (93%-100%) compared to radiosurgery (89%-96%), within the <2.5 cm tumor group included in this review. Radiosurgery consistently showed better hearing preservation (57%-68% versus 0%-14% for microsurgery). Vertigo and tinnitus outcomes generally favored microsurgery, while facial nerve outcomes were comparable across treatments. Follow-up durations ranged from approximately 36 to 88 months. 

Discussion

VS, also known as acoustic neuromas, are benign tumors that develop from the Schwann cells of the vestibular branch of the eighth cranial nerve. These tumors represent between 85% and 90% of intracranial growth in the cerebellopontine angle and are considered the third most common benign intracranial tumor [1]. In advanced cases, these tumors have been associated with trigeminal and facial neuropathies, brainstem compression, and hydrocephalus [3,5]. Several therapeutic options exist, including conservative management, microsurgery, radiotherapy, and radiosurgery. The choice of optimal treatment depends on several factors, such as location, size, tumor growth rate, and the patient's clinical status [9-11].

This study presents a systematic review, conducted through July 2025, on the outcomes of microsurgery versus radiosurgery in the treatment of VS. It examines the long-term results of microsurgery and radiosurgery to determine which therapeutic option yields the best outcomes based on clinical presentation of the participants, such as hearing loss, persistent tinnitus, vertigo, headaches, visual disturbances, ear pain, and facial paresthesia. The primary objective is to evaluate which method most effectively achieves complete tumor resection while preserving neurological and auditory function.

Four studies were assessed for methodological quality, encompassing a sample size of 1,167 participants. Participants were over 18 years of age, had tumors smaller than 2.5 cm, and were evaluated using standardized scales such as the Gardner-Robertson for hearing and the Dizziness Handicap Inventory for vertigo, allowing for a more uniform comparison of outcomes.

Tatagiba et al. and Pollock et al. concluded that microsurgery offers superior tumor control, within the size evaluated (<2.5 cm), suggesting a more favorable long-term outcome with surgical resection [17,20]. Although original studies discussed outcomes in larger tumors, these were not included in our review. While both Pollock et al. and Karpinos et al. found no statistically significant differences in tumor control between microsurgery and radiosurgery, Pollock et al. observed a slight trend favoring microsurgery, possibly due to direct removal of tumor tissue, which may reduce the risk of residual growth [19,20]. Overall, the available evidence indicates a consistent pattern in which microsurgery provides the most reliable tumor control across varying tumor sizes, while radiosurgery offers acceptable control for small lesions.

In contrast, when evaluating hearing preservation, the evidence consistently favors radiosurgery. Studies by Tatagiba et al., Myrseth et al., Karpinos et al., and Pollock et al. all reported better hearing outcomes in patients treated with radiosurgery compared to those who underwent microsurgery [17-20]. This advantage is likely due to the minimally invasive nature of radiosurgery, which minimizes the risk of mechanical or thermal damage to nearby auditory and neurological structures, thereby enhancing functional preservation. It is important to note that in the included studies, the middle fossa approach, which is generally considered the preferred microsurgical technique for hearing preservation in small intracanalicular tumors, was almost never used. As a result, the hearing outcomes reported here primarily reflect retrosigmoid surgery and may not fully represent the audiological results achievable with the middle fossa approach. These physiological differences highlight why radiosurgery generally preserves hearing, the cochlear nerve is not manipulated, and vascular supply to the inner ear remains undisturbed. However, microsurgical manipulation, even when carefully performed, poses a higher risk of compromising cochlear nerve integrity. 

Regarding vestibular symptoms such as tinnitus and vertigo, most findings appear to support microsurgery. Tatagiba et al., Myrseth et al., and Karpinos et al. observed greater improvement in these symptoms following surgical resection, likely due to the immediate decompression effect achieved through tumor removal [17-19]. However, Pollock et al. reported that patients treated with radiosurgery experienced a more favorable subjective perception of postural balance, indicating that this technique may, in some cases, promote better functional adaptation [20]. This pattern suggests that removing the tumor can relieve pressure on vestibular structures, explaining the improvements in dizziness and tinnitus after microsurgery, whereas radiosurgery may allow the vestibular system to adapt gradually through central compensation. It should be noted that reductions in tinnitus after microsurgery may partly reflect postoperative hearing loss rather than true symptom improvement, as tinnitus often diminishes when cochlear nerve function is severely compromised. These symptom comparisons must be interpreted within the context of small tumors (<2.5 cm), as larger lesions typically present a different vestibular burden. 

Quality of life (QoL) outcomes were reported in only one of the included studies. Pollock et al. evaluated QoL using the Health Status Questionnaire (HSQ) and found a decline in several physical functioning domains following microsurgical resection, whereas the radiosurgery group did not experience a significant decline during follow-up. Their study also noted that patient satisfaction was higher in the radiosurgery cohort, largely due to better preservation of hearing and facial nerve function. Because QoL metrics were not consistently assessed across the remaining studies, these findings cannot be generalized. However, they highlight the importance of incorporating standardized QoL measures in future comparative research. 

Nonetheless, several limitations should be acknowledged, including the lack of randomized controlled trials (RCTs) directly comparing microsurgery versus radiosurgery. Because the included studies consisted of both retrospective and prospective cohorts, differences in study design may influence the reported outcomes, particularly due to variability in data collection and risk of bias. The predominance of retrospective study designs may introduce selection bias. Additionally, variability in follow-up durations and in the criteria used to assess clinical outcomes complicates direct comparison between techniques. The exclusion of studies published in languages other than English and those with incomplete data may also limit the generalizability of the findings. Other methodological limitations include the inherent heterogeneity among the included cohorts, the absence of blinding in outcomes assessment, and differences in how hearing and vestibular outcomes were defined across studies. The definition of long-term follow-up also varied widely across the included studies, ranging from approximately 2 to 15 years, which affects the comparability of the reported outcomes. Cost-effectiveness and long-term recurrence risk were also not assessed, despite their relevance to treatment planning. These findings apply only to VS smaller than 2.5 cm, as larger tumors may demonstrate different tumor control and functional outcomes. Therefore, any references in the original studies to outcomes in larger tumors cannot be generalized to this review. In all included studies, microsurgery was performed with the intention of achieving gross total resection, and none reported planned subtotal resections. Therefore, the tumor control outcomes reflect complete removal rather than residual-tumor management strategies.

Both microsurgery and radiosurgery are valid and safe options for the treatment of VS. The choice should be individualized, considering tumor size, the patient's functional status, and personal preference. Microsurgery offers better results in tumor control and improvement of vestibular symptoms, while radiosurgery is superior in hearing preservation. Further prospective studies evaluating QoL and long-term functional outcomes are recommended to optimize clinical decision-making.

## Conclusions

This systematic review compared microsurgery and radiosurgery in the management of VS and found that microsurgery offers higher tumor control rates (93%-100%) within tumors smaller than 2.5 cm and is more effective in improving symptoms such as vertigo and tinnitus. Meanwhile, radiosurgery demonstrates superior hearing preservation (57%-68%), particularly in patients with small tumors and pre-treatment serviceable hearing.

These findings represent overall trends from the included observational studies and may vary on a case-by-case basis. In conclusion, both techniques are effective and safe, and treatment decisions should be individualized according to tumor size (<2.5 cm), clinical symptoms, hearing status, and patient preference. Because all available evidence comes exclusively from observational studies, the results should be interpreted with caution. Further prospective randomized trials are necessary to strengthen the current evidence and enhance the rigor of future studies.
